# Post-Traumatic Pseudoaneurysm of the Superficial Temporal Artery in a Pediatric Patient

**DOI:** 10.5334/jbsr.3024

**Published:** 2023-01-02

**Authors:** Martine Leeman, Astrid Leus, Caroline Ernst

**Affiliations:** 1 UZ Brussel, BE

**Keywords:** Pseudoaneurysm, yin-yang sign, superficial temporal artery, head injury, child

## Abstract

**Teaching Point:** A pseudoaneurysm of the superficial temporal artery is a rare complication of head injury in a child.

## Case History

A 14-year-old boy was admitted to the emergency department after a road traffic accident in which he suffered from a head injury, stiff neck, and multiple abrasions. A hematoma and tiny laceration were visible in the right anterior temporal region.

**Figure 1:**
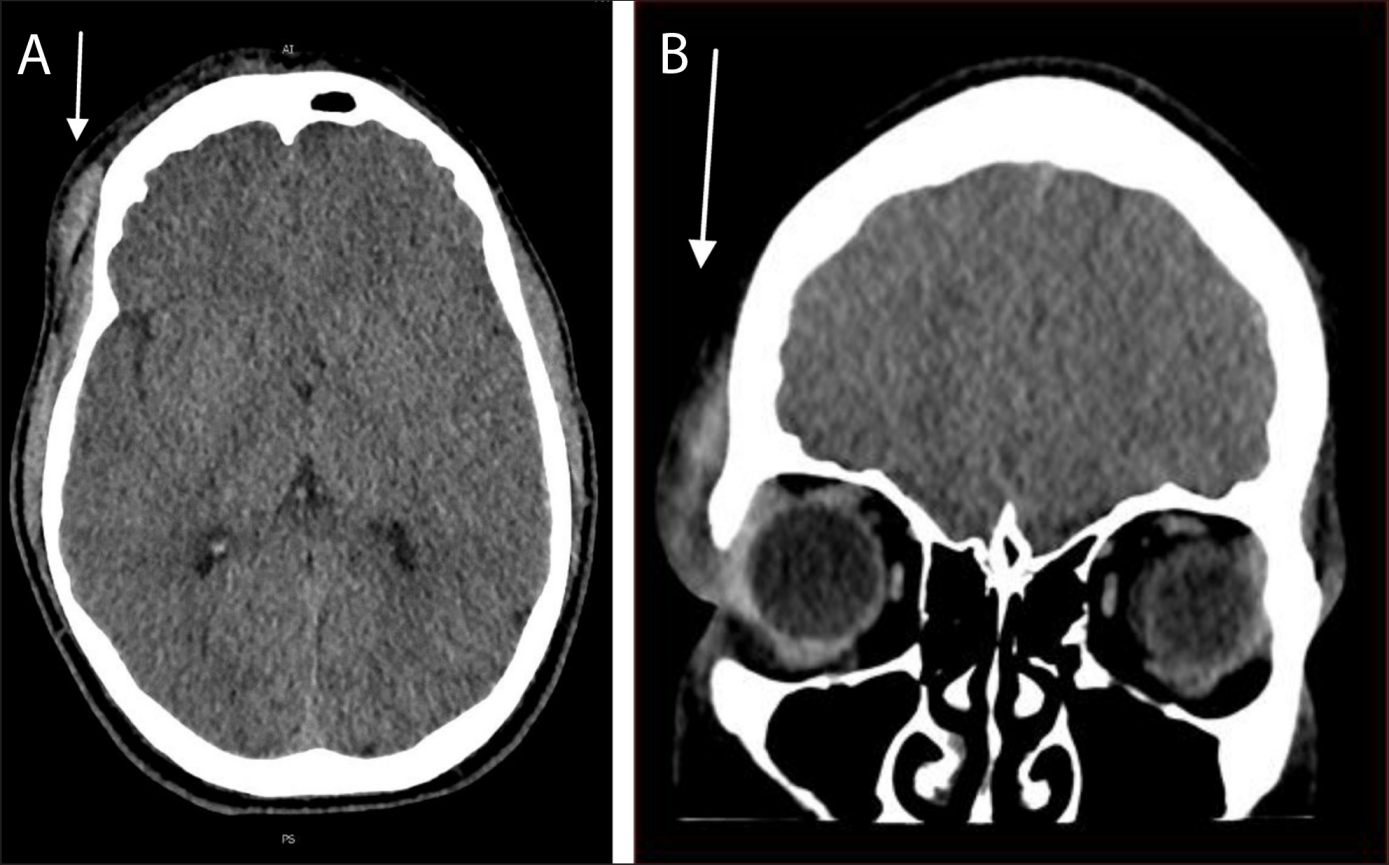


Computer tomography (CT) of the brain and spine showed a supra-orbital scalp hematoma on the right side (Figure 1A–B). No fractures could be detected.

**Figure 2:**
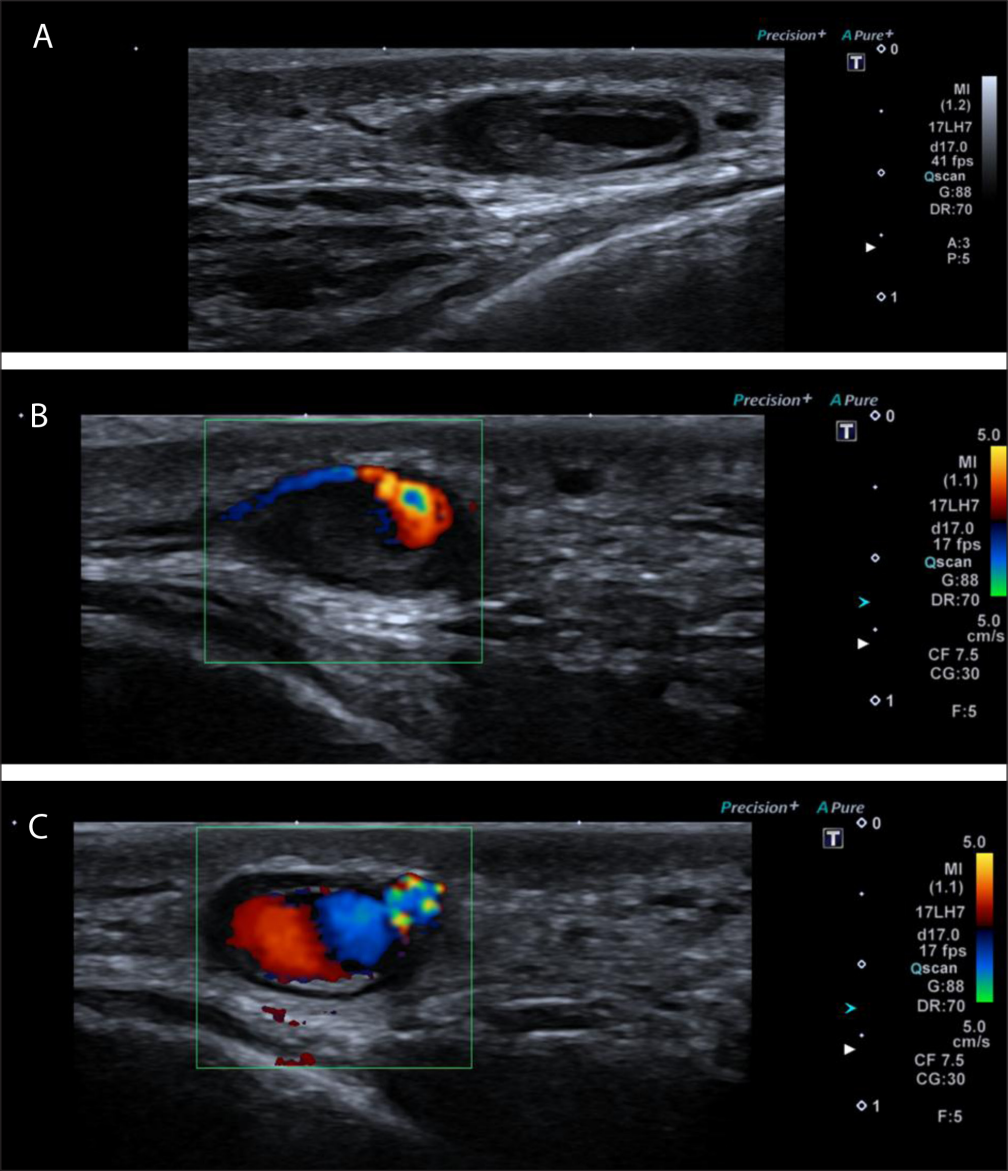


Seven weeks post trauma, the boy presented with a slow growing, pulsatile, compressible, painless mass in the right temporal area. B-mode ultrasound revealed a 1.1 × 0.9 × 0.5 cm saccular central hypoechoic structure with peripheral hyperechogenicity (Figure 2A). Color-Doppler ultrasound (CDUS) revealed a vascular structure associated with and displacing the superficial temporal artery (Figure 2B) with an embedded yin-yang sign suggestive of ‘to-and-fro flow’ (Figure 2C). The image was compatible with a partially thrombosed pseudoaneurysm of the superficial temporal artery.

The patient was referred to interventional radiology for treatment by compression. However, because compression was unsuccessful, the child had to undergo surgical resection of the lesion with ligation of the proximal and distal ends of the superficial temporal artery.

### Comments

Pseudoaneurysms of the superficial temporal artery are very rare, particularly in children. The most common cause is blunt trauma from motor vehicle accidents or sports-related injuries. Lesser common causes are penetrating injuries from lacerations, gun shot or stab wounds and surgery. Iatrogenic causes such as neurosurgical procedures or hair transplantation were also reported [1]. Since children seldom get involved in this type of accidents, it may explain the rarity in children.

The anterior division of the superficial temporal artery is most vulnerable to trauma and development of a pseudoaneurysm since there is no cushioning by the frontalis and temporalis muscle. Patients usually present with a painless, pulsatile, compressible mass in the frontotemporal region with thrill on auscultation. Pulsation may be absent in case of complete thrombosis. Differential diagnosis includes abscess, lipoma, cyst, hematoma, lymph node, neuroma of the n. supra-orbitalis, angiofibroma, arteriovenous fistula, meningocele and encephalocele. In most cases, particularly in children, CDUS is the imaging modality of choice over CT angiography, so the use of contrast and radiation can be avoided. Typical image on B-mode ultrasound is the ‘black hole sign’. Doppler ultrasound demonstrates a bidirectional swirling pattern of blood flow in the lesion known as yin-yang sign.

Given the risk of rupture and hemorrhage, treatment is recommended. Possible treatments include compression, embolization and resection.
